# Technology CAD (TCAD) Simulations of Mg_2_Si/Si Heterojunction Photodetector Based on the Thickness Effect

**DOI:** 10.3390/s21165559

**Published:** 2021-08-18

**Authors:** Hong Yu, Shentong Ji, Xiangyan Luo, Quan Xie

**Affiliations:** 1 The College of Big Data and Information Engineering, Guizhou University, Guiyang 550025, China; yuhong@gznc.edu.cn (H.Y.); gz.xyluo17@gzu.edu.cn (X.L.); 2 The College of Physics and Electronic Science, Guizhou Education University, Guiyang 550018, China; jishentong@gznc.edu.cn

**Keywords:** thickness, Mg_2_Si/Si heterojunction, PD, optical and electrical properties, Silvaco TCAD

## Abstract

Research on infrared detectors has been widely reported in the literature. For infrared detectors, PbS, InGaAs, PbSe, InSb, and HgxCd1-xTe materials are the most widely used and have been explored for photodetection applications. However, these are toxic and harmful substances which are not conducive to the sustainable development of infrared detectors and are not eco-friendly. Mg_2_Si is a green, healthy, and sustainable semiconductor material that has the potential to replace these toxic and damaging photoelectric materials, making photoelectric detectors (PDs) green, healthy, and sustainable. In this work, we report on the results of our simulation studies on the PN junction Mg_2_Si/Si heterojunction PD. A model structure of Mg_2_Si/Si heterojunction PD has been built. The effects of Mg_2_Si and Si layer thickness on the optical and electrical performance of Mg_2_Si/Si heterojunction PD are discussed. For the purpose of this analysis, we consider electrical performance parameters such as I–V curve, external quantum efficiency (EQE), responsivity, noise equivalent power (NEP), detectivity, on-off ratio, response time, and recovery time. The simulation results show that the Mg_2_Si/Si heterojunction PD shows optimum performance when the thickness of Si and Mg_2_Si layers are 300 nm and 280 nm, respectively. For the optimized structure, the reverse breakdown voltage was found to be −23.61 V, the forward conduction voltage was 0.51 V, the dark current was 5.58 × 10^−13^ A, and the EQE was 88.98%. The responsivity was found to be 0.437 A/W, the NEP was 6.38 × 10^−12^ WHz^1/2^, and the detectivity was 1.567 × 10^11^ Jones. With the on-off ratio of 1566, the response time was found to be 0.76 ns and the recovery time was 5.75 ns. The EQE and responsivity peak wavelength of PD show a redshift as the thickness of Mg_2_Si increases. The Mg_2_Si heterojunction PD can effectively detect infrared light in the wavelength range of 400 to 1400 nm. The simulation results can be utilized to drive the development of green Mg_2_Si/Si heterojunction PD in the future.

## 1. Introduction

Telecommunications, healthcare, security and safety, aerospace, and automobile night vision systems are just a few of the commercial uses for infrared photodiodes that have recently attracted special attention. In comparison to Si photodiodes, Mg_2_Si/Si photodiodes show a spectral response with a longer cutoff wavelength and higher intensity around the photon energy threshold [1]. Mg_2_Si is an indirect bandgap, environmentally friendly semiconductor material which has been extensively studied for use as thermoelectric material [2], battery material [3,4], structural material [5], and composite material [6]. Mg and Si are abundant in nature, non-toxic, and pollution-free, and have eco-friendly characteristics [7]. It has a bandgap of 0.6–0.8 eV and a high absorption coefficient of more than 10^5^ cm^−1^ around 500 nm [8]. Mg_2_Si has recently attracted attention as a suitable candidate for short-wavelength IR(SWIR) sensors. Mg_2_Si/Si could be used as a safe alternative to the poisonous sensors already in use for night vision and SWIR light detection. Due to Mg_2_Si’s unique features for IR sensitivity, there are a few reports of such materials being used in IR sensor applications [1,9]. Using magnetron sputtering and thermal evaporation, our research group has previously reported on environmentally friendly Mg_2_Si semiconductor thin films [10,11,12]. In this work, we extend our work towards the modeling of p-Mg_2_Si/n-Si heterojunction PD using the Atlas module of Silvaco TCAD software. The effects of different Mg_2_Si and Si thicknesses on the I–V curve, EQE, responsivity, NEP, detectivity, on-off ratio, response time, and recovery time of Mg_2_Si/Si heterojunction PD are studied, and the simulation results are examined and analyzed. The simulation results are used to create the model. The Mg_2_Si heterojunction PD can effectively detect infrared light in the wavelength range of 400 to 1400 nm. Our results indicate that the Mg_2_Si has significant research value as a potential candidate material for infrared sensing and night vision applications. The simulation results can be utilized for the development of a green Mg_2_Si/Si heterojunction PD in the future.

## 2. Method and Model

Silvaco TCAD’s Atlas device simulator module software can mimic the electrical, optical, and thermal characteristics of semiconductor devices. Atlas is a simple and extensible platform based on physical modularization that analyses the AC, DC, and time-domain responses of 2D and 3D devices, as well as the properties of opto-electric and electro-optical conversion. The device structure is generated by the device editor or process simulation. After describing the material parameters, physical model, electrical contact type, and calculation method in the device, the device structure’s characteristics can be calculated by Atlas. Atlas allows users to input material parameters, such as energy band parameters, dielectric constant, lifetime parameters, mobility parameters, etc. It can also customize the material and change the physical model, all of which provide a technique for simulation calibration.

**Figure 1:**
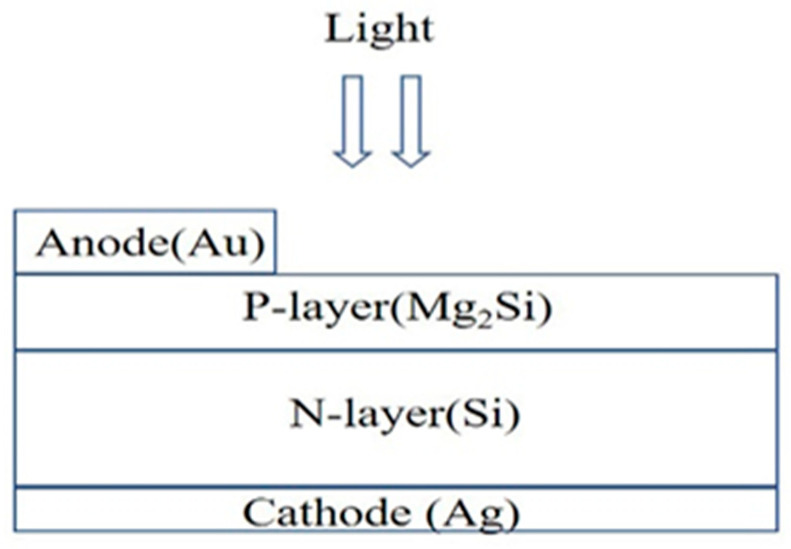
The schematic diagram of the Mg_2_Si/Si heterojunction PD.

**Figure 2:**
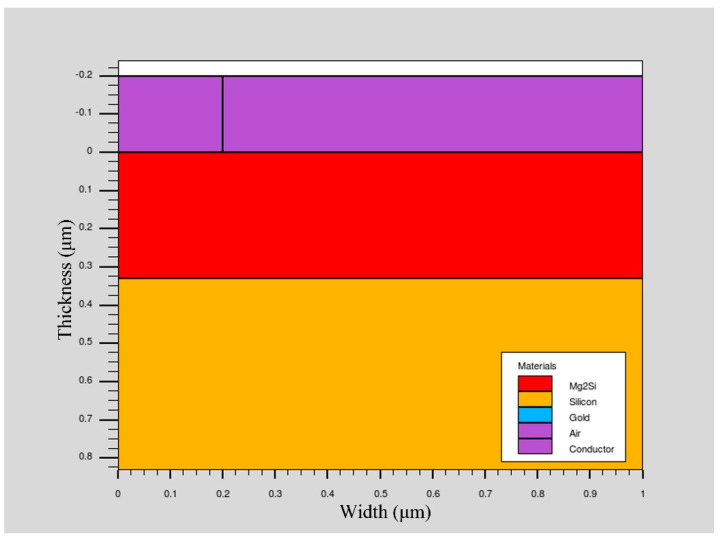
The cross–section of the Mg_2_SiSi heterojunction PD.

**Figure 3:**
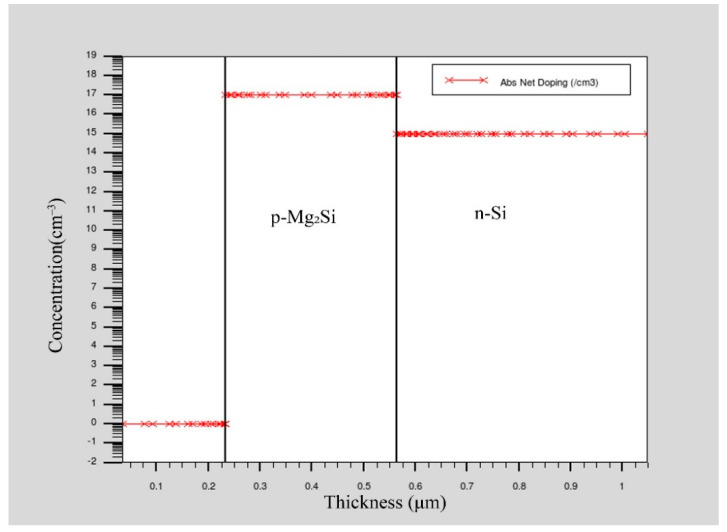
The concentration distribution of Mg_2_Si/Si heterojunction PD.

In the simulation process, the p-Mg_2_Si/n-Si heterojunction PD structure was established on the two-dimensional grid. The schematic diagram is shown in Figure 1. The region, material, doping, and electrode are defined in turn. SRH recombination model, Auger recombination model, conmob mobility model, fldmob electric field dependent model, and Selberherr impact ionization model were selected. A Newton iterative algorithm was used to solve the Poisson and continuity equations, and then the solution of the device was obtained and the optoelectronic characteristics of the device were analyzed. The doping concentration of p-Mg_2_Si was 1 × 10^17^ cm^−3^, the width was 1 μm, and the thicknesses considered in the simulation were 0.08 μm, 0.13 μm, 0.18 μm, 0.23 μm, 0.28 μm, 0.33 μm, and 0.38 μm, respectively. The doping concentration of n-Si was 1 × 10^15^ cm^−3^, the width was 1 μm, and the thicknesses considered in the simulation were 0.3 μm, 0.4 μm, 0.5 μm, 0.6 μm, and 0.7 μm, respectively. The anode material was gold (Au), with a width of 0.2 μm and a thickness of 0.2 μm. The cathode material was silver (Ag), with a length of 1 μm and a thickness of 0.2 μm. The Au and Ag electrodes increase the film’s average absorption and forward scattering over a broad spectrum, thus significantly reducing its total reflection performance [13,14]. The optical and electrical material parameters for Mg_2_Si and Si used in the simulation are shown in Table 1 [15,16,17,18]. The cross-section of the Mg_2_Si/Si heterojunction PD is shown in Figure 2. The concentration distribution of Mg_2_Si/Si heterojunction PD is shown in Figure 3.

## 3. Results and Discussion

### 3.1. I–V Curves

**Figure 4:**
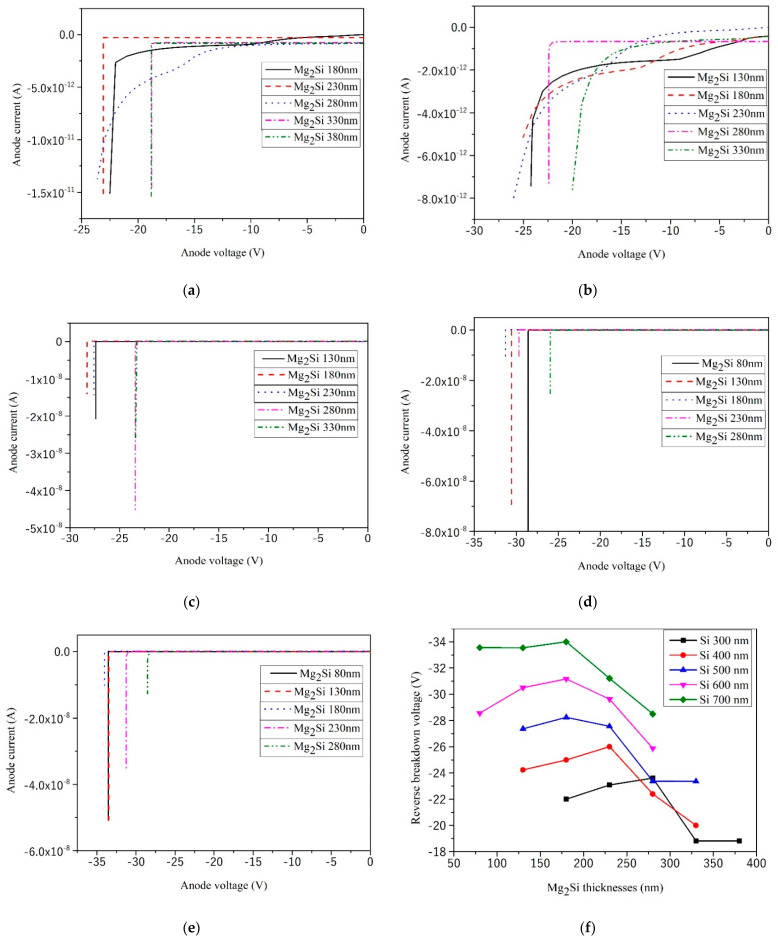
The reverse I–V curves of the heterojunction PD with different Mg_2_Si thicknesses under the condition of 300 nm, 400 nm, 500 nm, 600 nm, and 700 nm thicknesses of Si. (**a**) Si-300 nm; (**b**) Si-400 nm; (**c**) Si-500 nm; (**d**) Si-600 nm; (**e**) Si-700 nm; (**f**) The effect of thicknesses on the reverse breakdown voltage.

**Figure 5:**
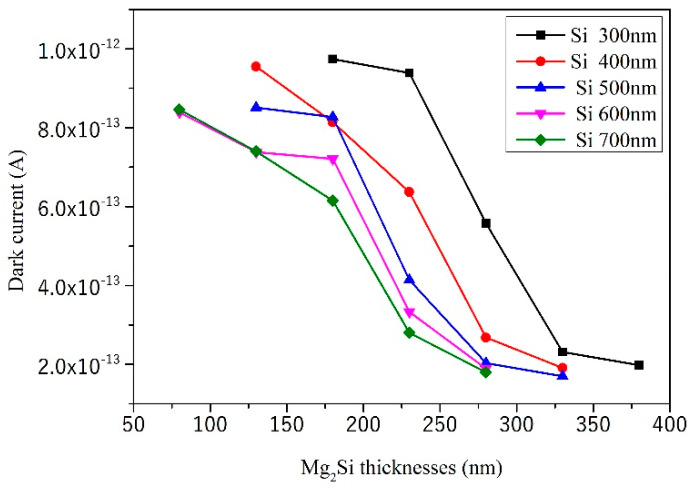
The effect of thicknesses on the dark current.

First, without illumination, the heterojunction PD was reversely biased. The simulated results of the reverse I–V curves of the heterojunction PD with different Mg_2_Si thicknesses under the condition of 300 nm, 400 nm, 500 nm, 600 nm, and 700 nm Si thicknesses are shown in Figure 4. The thicknesses of Mg_2_Si were increased from 80 nm to 380 nm, respectively. The effect of thicknesses on the reverse breakdown voltage of heterojunction PD is shown in Figure 4f. The effect of thicknesses on dark current of heterojunction PD is shown in Figure 5.

The direction of the built-in electric field of p-Mg_2_Si/n-Si PD is from the Si layer to Mg_2_Si. It can be seen from Figure 4 that with the increase in Mg_2_Si thickness, the reverse breakdown voltage of Mg_2_Si/Si heterojunction PD first increases and then decreases. The thickness of the Mg_2_Si layer corresponding to the peak value of the reverse breakdown voltage is Wpmax. To improve the reverse voltage endurance of the heterojunction, the thickness of the absorption layer can be increased to avoid the punch-through between the space charge region and the electrode under higher reverse bias. Wpmax is the critical thickness of punch-through between the space charge region and the electrode of the heterojunction PD, which is about 200 nm. When Wp < Wpmax, the space charge region and the electrode are in punch-through states, resulting in the widening of the depletion region. The avalanche effect increases, so the reverse breakdown voltage increases. When Wp > Wpmax, the space charge region and the electrode cannot be punched through, resulting in a narrowing of the depletion region. The avalanche effect is reduced, so the reverse breakdown voltage is reduced.

**Figure 6:**
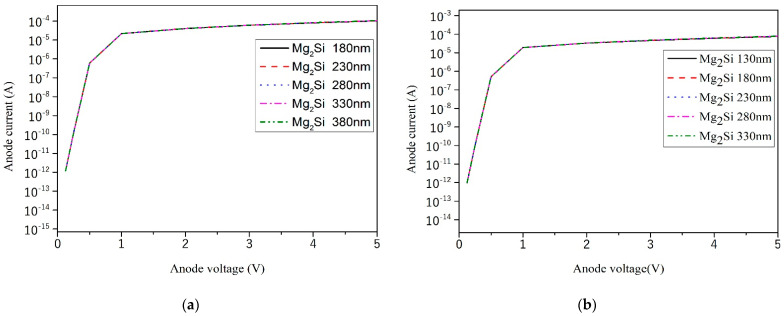

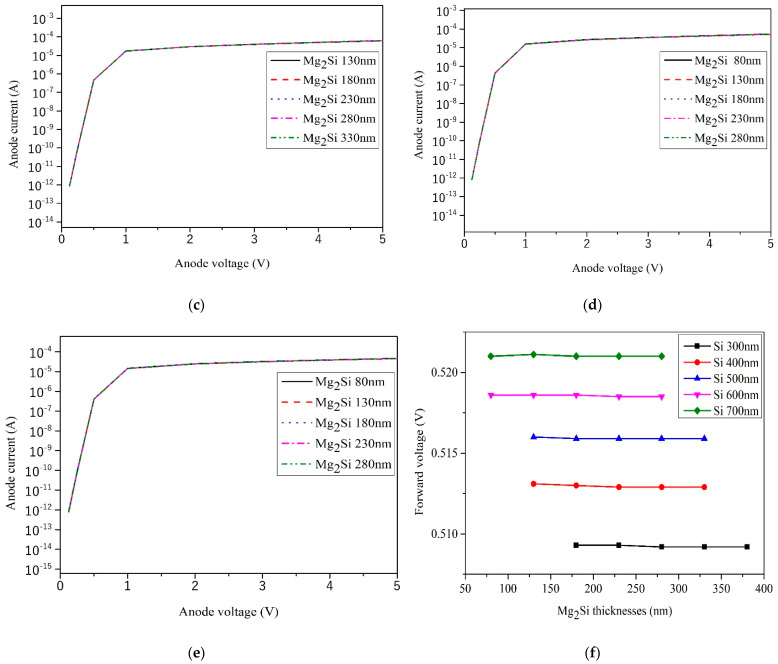
The forward I–V curves of the heterojunction PD with different Mg_2_Si thicknesses of 300 nm, 400 nm, 500 nm, 600 nm, and 700 nm Si thicknesses were plotted on a semilog scale. (**a**) Si-300 nm; (**b**) Si-400 nm; (**c**) Si-500 nm; (**d**) Si-600 nm; (**e**) Si-700 nm; (**f**) The effect of thicknesses on the forward voltage.

The simulation results of forward I–V curves when the Mg_2_Si/Si heterojunction PD was forward biased are shown in Figure 6. With the increase in Mg_2_Si thickness, the forward conduction voltage of Mg_2_Si/Si heterojunction PD was almost stable in the voltage range of 0.51–0.52 V. The forward voltage of the heterojunction does not change with the thickness of Mg_2_Si. Its value is less than the forward conduction voltage of ordinary silicon diode 0.6–0.8 V.

### 3.2. EQE

The quantum efficiency can be divided into EQE and internal quantum efficiency (IEQ). In this paper, the quantum efficiency refers to the EQE, that is, the number of charge carriers generated by each incident photon. The quantum efficiency reflects the sensitivity of the photoelectric device to photons and can be expressed as [19]:

(1) $$\mathrm{EQE}=\frac{I_{p}}{q\phi }=\frac{I_{p}}{q}\frac{h\nu }{p}\times 100\%$$

**Figure 7:**
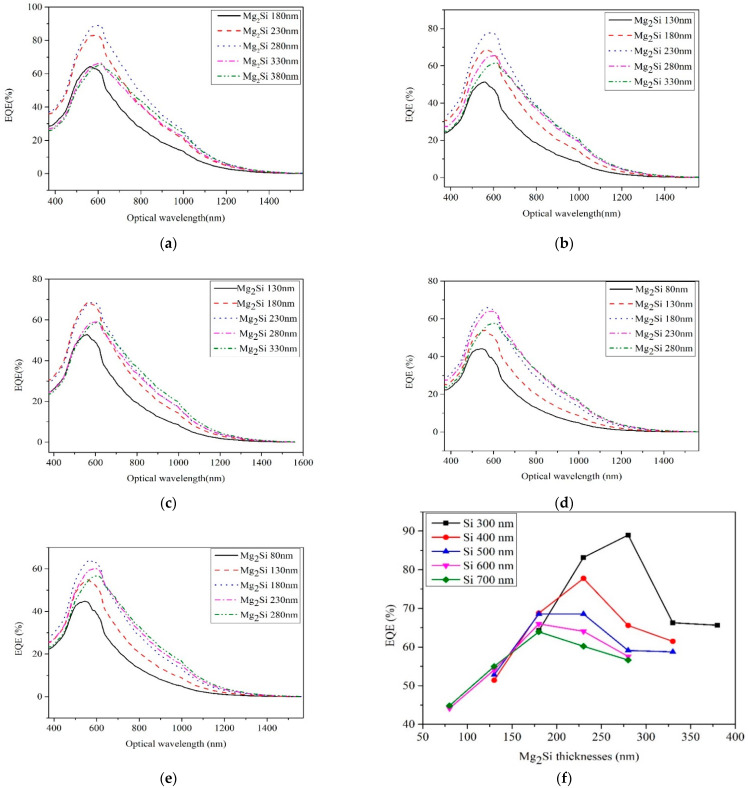
The EQE curves of heterojunction PD with different thicknesses of Mg_2_Si under the conditions of Si thicknesses of 300 nm, 400 nm, 500 nm, 600 nm, and 700 nm. (**a**) Si-300 nm; (**b**) Si-400 nm; (**c**) Si-500 nm; (**d**) Si-600 nm; (**e**) Si-700 nm; (**f**) The effect of thicknesses on the EQE.

In the equation, *I_p_* is the average optical current output of the heterojunction PD, *ϕ* = *p*/*hν* is the optical flux, and *p* is the incident optical power. Figure 7 shows the EQE curves of heterojunction PD with different thicknesses of Mg_2_Si under the conditions of Si thicknesses of 300 nm, 400 nm, 500 nm, 600 nm, and 700 nm, respectively. The effect of thicknesses on EQE of heterojunction PD is shown in Figure 7f. When the thicknesses of the Si and Mg_2_Si layers are 300 nm and 280 nm, respectively, the EQE of the Mg_2_Si/Si heterojunction PD reached the maximum of 88.98%. Sharma et al. [20] calculated the photoelectric properties of ZnO/Si heterojunction diodes and obtained 93% of the EQE, which is equivalent to the maximum EQE in this paper.

In the beginning, with the increase in Mg_2_Si thickness, the Mg_2_Si layer provides more photogenerated carriers which are conducive to the improvement of EQE. However, with the further increase in Mg_2_Si thickness, more photogenerated carriers are concentrated near the surface of the Mg_2_Si layer, and there is not enough photon energy to reach the interface of the Mg_2_Si/Si heterojunction PD so that the EQE of the device decreases. The EQE peak wavelength of PD shows a redshift as the thickness of Mg_2_Si increases. When the Mg_2_Si layer is thin, most of the long wavelengths pass directly through the Mg_2_Si absorption layer without absorption. At this time, these long wavelengths of light do not contribute to the generation of photogenerated carriers. Longer wavelength light needs to penetrate a greater depth to be fully absorbed [21]. Some longer wavelength light is absorbed as the thickness of the Mg_2_Si layer increases, so the EQE peak wavelength of PD shows a redshift as the thickness of Mg_2_Si increases. The peak EQE of heterojunction PD with different Mg_2_Si thickness decreases when the thickness of Si increases because the increase in the thickness of Si substrate hinders the transport of part of the photogenerated carriers.

### 3.3. Responsivity

**Figure 8:**
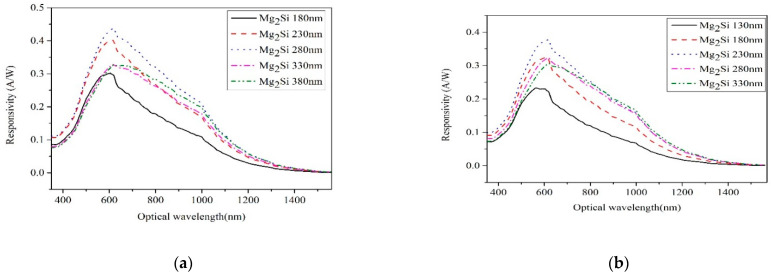

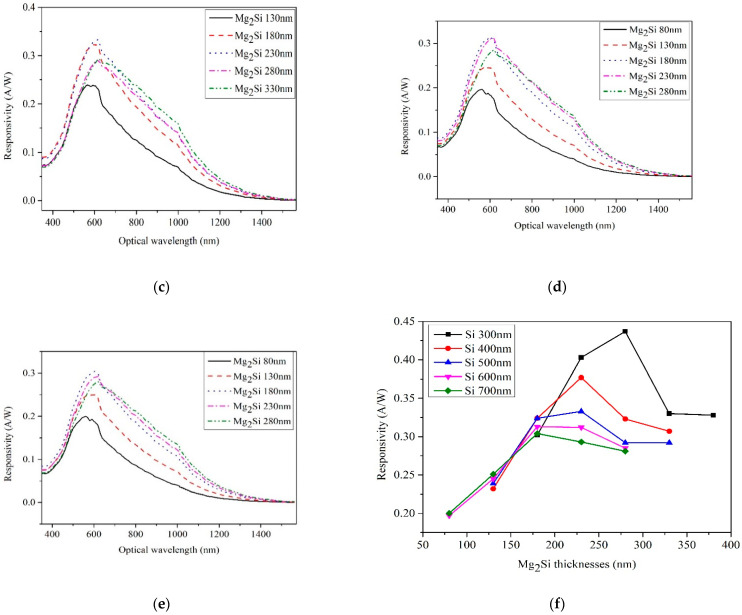
The responsivity curves of heterojunction PD with different thicknesses of Mg_2_Si under the conditions of Si thicknesses of 300 nm, 400 nm, 500 nm, 600 nm, and 700 nm. (**a**) Si-300 nm; (**b**) Si-400 nm; (**c**) Si-500 nm; (**d**) Si-600 nm; (**e**) Si-700 nm; (**f**) The effect of thicknesses on the responsivity.

Figure 8 shows the responsivity curves of heterojunction PD with different thicknesses of Mg_2_Si under the conditions of Si thicknesses of 300 nm, 400 nm, 500 nm, 600 nm, and 700 nm, respectively. The responsivity of a heterojunction PD is the ratio of the output signal to the incident light power. It is defined as the ratio of the heterojunction PD’s output photocurrent to the incident light power P under incident light illumination, which may be represented as [22]:

(2) $$\mathrm{Responsivity}=\frac{I_{p}}{P}=\frac{q\eta \lambda }{hc}=\frac{\eta \lambda \;\left(\mu m\right)}{1.24}$$

In the formula, *λ* is the wavelength (μm) and *η* is the EQE. The effect of thicknesses on responsivity of heterojunction PD is shown in Figure 8f. The responsivity of Mg_2_Si/Si heterojunction PD reached the maximum value of 0.437 A/W when the thickness of Si and Mg_2_Si layers were 300 nm and 280 nm, respectively. Liu et al. [23] reported a p-ZnO/n-Si heterojunction PD and obtained a responsivity value of 0.206 A/W. The responsivity peak wavelength of PD shows a redshift as the thickness of Mg_2_Si increases.

The Mg_2_Si layer supplies more photogenerated carriers and transfers them to the Si layer as the thickness of the Mg_2_Si layer increased, thus increasing the photocurrent and responsivity. The more photogenerated carriers are concentrated on the surface of the Mg_2_Si layer, the more difficult they are to collect by an electric field, resulting in a decrease in photocurrent and responsivity as the thickness of the Mg_2_Si layer increases. Only the carriers for a particular thickness near the Mg_2_Si/Si interface are injected into the circuit under the action of the built-in electric field. When the thickness reaches a given level, the carriers far away from the interface are compounded before reaching the Mg_2_Si/Si interface [24]. Therefore, when the thickness of the Mg_2_Si layer continues to increase, the current response and responsivity of the heterojunction do not change much. If the thickness of the Mg_2_Si layer is too thick, most of the light energy is absorbed by the Mg_2_Si layer and there is not enough photon energy to reach the surface of the Mg_2_Si/Si heterojunction which reduces the response current of the heterojunction.

When the wavelength of incident light was increased from 400 nm to 1400 nm, the responsivity first increased and then decreased with the increase in wavelength. The responsivity reaches its maximum near the incident light at 600 nm. The shorter the wavelength, the more energy it has. Therefore, high-energy photons will lose a lot of energy as they strike the device’s surface, and absorption by the device’s front surface will be considerably reduced, resulting in a reduction in current responsiveness and EQE [25]. When the wavelength is larger than 600 nm, the quantum efficiency and responsivity decrease rapidly because the Mg_2_Si layer absorbs less light at long wavelengths. If the diffusion length of carriers is too long, they may be recombined before they are separated by the built-in electric field, thus reducing the current response.

### 3.4. NEP

The NEP is defined as the incident light power within the 1 Hz output bandwidth when the signal-to-noise ratio of the device is equal to 1. It is shown as follows [26]:

(3) $$\mathrm{NEP}=\frac{\sqrt{\frac{I_{noise}^{2}}{1\;\mathrm{Hz}}}}{R}$$

**Figure 9:**
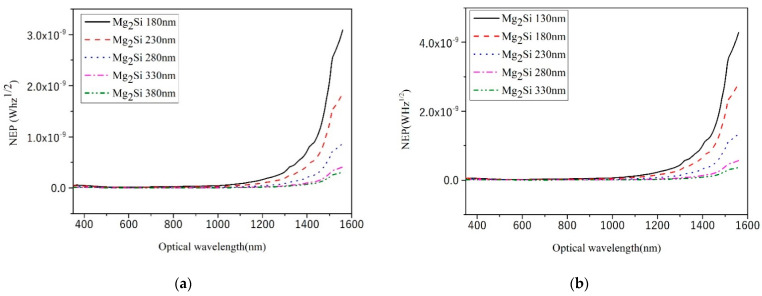

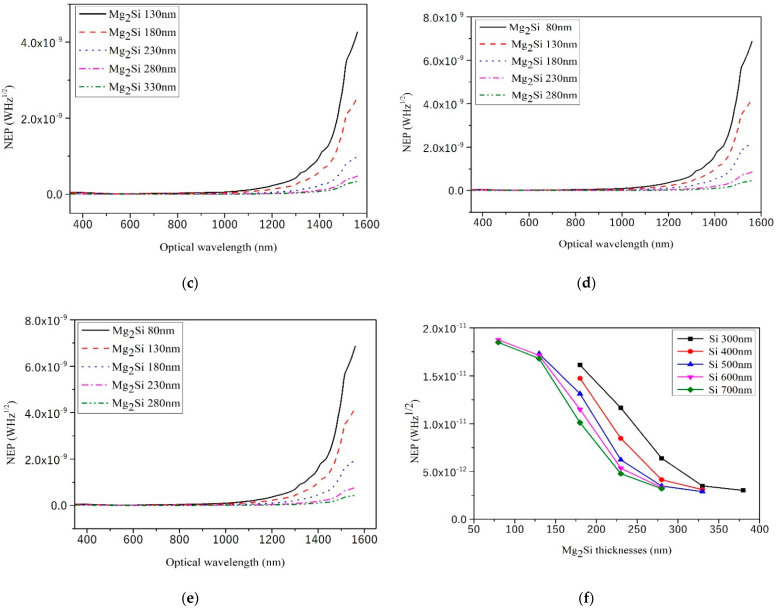
The NEP curves of heterojunction PD with different Mg_2_Si thicknesses under the condition of 300 nm, 400 nm, 500 nm, 600 nm, and 700 nm thicknesses of Si. (**a**) Si-300 nm; (**b**) Si-400 nm; (**c**) Si-500 nm; (**d**) Si-600 nm; (**e**) Si-700 nm; (**f**) The effect of thicknesses on the NEP.

In the formula, NEP is the noise equivalent power and the unit is WHz^1/2^. *I_noise_* is the noise current. This article only considers the noise caused by dark current. *R* is the responsivity. The smaller the NEP, the smaller the incident light power effectively detected by the device and the better the detector performance. The NEP curves of heterojunction PD with different Mg_2_Si thicknesses are shown in Figure 9 under the condition of 300 nm, 400 nm, 500 nm, 600 nm, 500 nm, and 700 nm thicknesses of Si, respectively. As shown in Figure 9f, the NEP values of Mg_2_Si/Si heterojunction PD with different thicknesses are 10^−11^–10^−12^ WHz^1/2^. An et al. [26] reported Graphene/Si heterojunctions and found that its minimum NEP was 0.92 × 10^−12^ WHz^1/2^.

When the thickness of the Si substrate is constant, the NEP value of heterojunction PD decreases with the increase in Mg_2_Si thickness. It can be seen from Equation (3) that when the responsivity value changes little with the increase in Mg_2_Si thickness, the NEP is related to the dark current value. Dark current mainly includes reverse saturation current, recombination current in the barrier region, and surface leakage current. In the case of no illumination and a small reverse bias voltage (−3 V), the increase in Mg_2_Si thickness effectively decreases the surface leakage current of the heterojunction [27], thus reducing the dark current and NEP.

### 3.5. Detectivity

**Figure 10:**
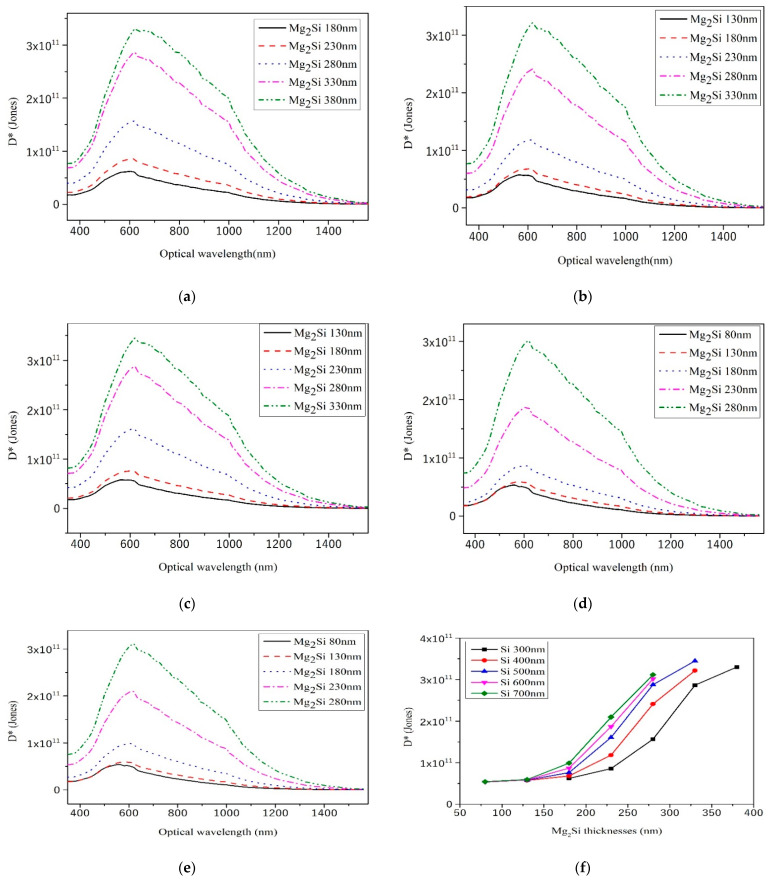
The D* curves of heterojunction PD with different Mg_2_Si thicknesses under the condition of 300 nm, 400 nm, 500 nm, 600 nm, and 700 nm thicknesses of Si. (**a**) Si-300 nm; (**b**) Si-400 nm; (**c**) Si-500 nm; (**d**) Si-600 nm; (**e**) Si-700 nm; (**f**) The effect of thicknesses on the D*.

The detectivity and NEP are reciprocal to each other. The higher the detectivity, the better the detection performance of the device. Considering the difference in the effective detection area of the device, the detection measure is defined as follows [28]:

(4) $$D^{*}=\frac{\sqrt{A}}{\mathrm{NEP}}$$

where D* is the detectivity and the unit is cmHz^1/2^W^−1^ (Jones). *A* is the effective working area of heterojunction PD and its unit is cm^2^. Figure 10 shows the D* curves of heterojunction PD with different thicknesses of Mg_2_Si for fixed thicknesses of Si at 300 nm, 400 nm, 500 nm, 600 nm, and 700 nm, respectively. As shown in Figure 10f, the D* values of Mg_2_Si/Si heterojunction PD with different thicknesses are 10^10^–10^11^ Jones. Xu et al. [29] reported SnS_2_/Si vertical heterostructure and obtained a maximum detectivity of ~10^11^ Jones. Hun et al. [30] fabricated a PD based on (CH_3_NH_3_)_3_Sb_2_Br_9_ (MA_3_Sb_2_Br_9_) and displayed a high detectivity of 4.32 × 10^11^ Jones.

When the thickness of the Si substrate is kept constant, the D* value of the heterojunction detector increases with the increase in Mg_2_Si layer thickness. It can be seen from Equation (4) that D* and NEP are reciprocal to each other. NEP value decreases with the increase in Mg_2_Si layer thickness, while the D* value increases with the increase in Mg_2_Si layer thickness.

### 3.6. On/Off Ratio

**Figure 11:**
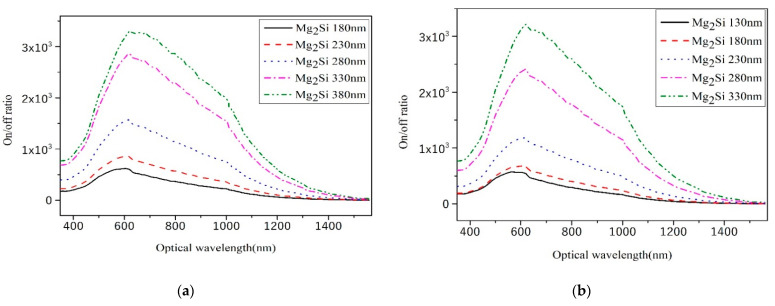

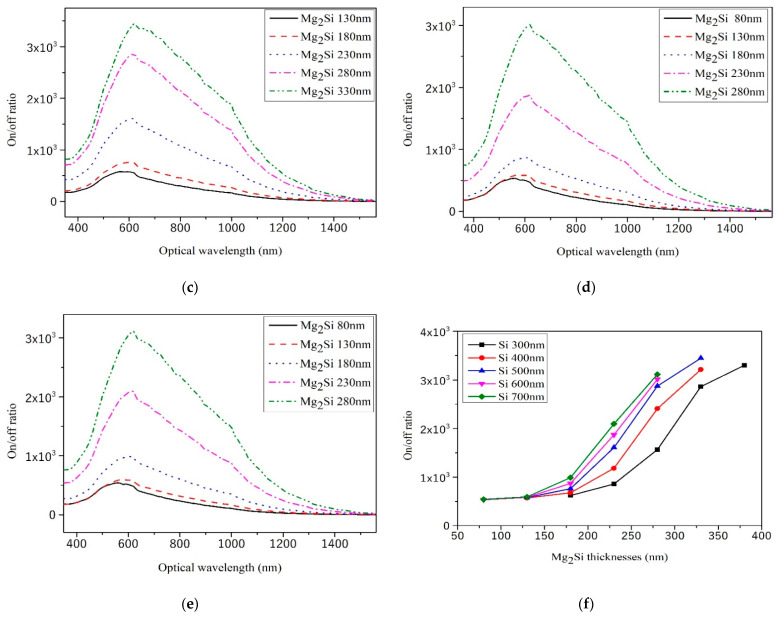
The on/off ratio curves of heterojunction PD with different Mg_2_Si thicknesses under the condition of 300 nm, 400 nm, 500 nm, 600 nm, and 700 nm thicknesses of Si. (**a**) Si-300 nm; (**b**) Si-400 nm; (**c**) Si-500 nm; (**d**) Si-600 nm; (**e**) Si-700 nm; (**f**) The effect of thicknesses on the on/off ratio.

The on/off ratio is the ratio of the output signal of the heterojunction PD with light and without light. This reflects the response properties of the heterojunction PD to incident light. The formula for the computation of the on/off ratio is as follows [31,32]:

(5) $$\mathrm{On}/\mathrm{off}\;\mathrm{ratio}=\frac{I_{light}}{I_{dark}}$$

where *I_light_* is the output current with illumination and *I_dark_* is the output current without illumination. Figure 11 shows the on/off curves of heterojunction PD with different thicknesses of Mg_2_Si for the thicknesses of Si at 300 nm, 400 nm, 500 nm, 600 nm, and 700 nm, respectively. The bias voltage was −25 V. As shown in Figure 11f, the on/off ratio values of Mg_2_Si/Si heterojunction PD with different thicknesses are 5.33 × 10^2^–3.45 × 10^3^ Jones. The CuO/Si NW array heterojunction PD were fabricated by Hong et al. [33], and a 10^2^ on/off ratio was obtained. Li et al. [34] fabricated InSb/Si heterojunction PD with an on/off ratio of 2.9 × 10^3^.

When the thickness of the Si substrate is kept constant, the on/off ratio value of the heterojunction detector increases with the increase in Mg_2_Si thickness. The photocurrent first increases and then decreases as the thickness of Mg_2_Si increases. The dark current decreases as the thickness of Mg_2_Si increases. It is observed from Equation (5) that the on/off ratio value increases with the increase in Mg_2_Si thickness.

### 3.7. Response Time and Recovery Time

**Figure 12:**
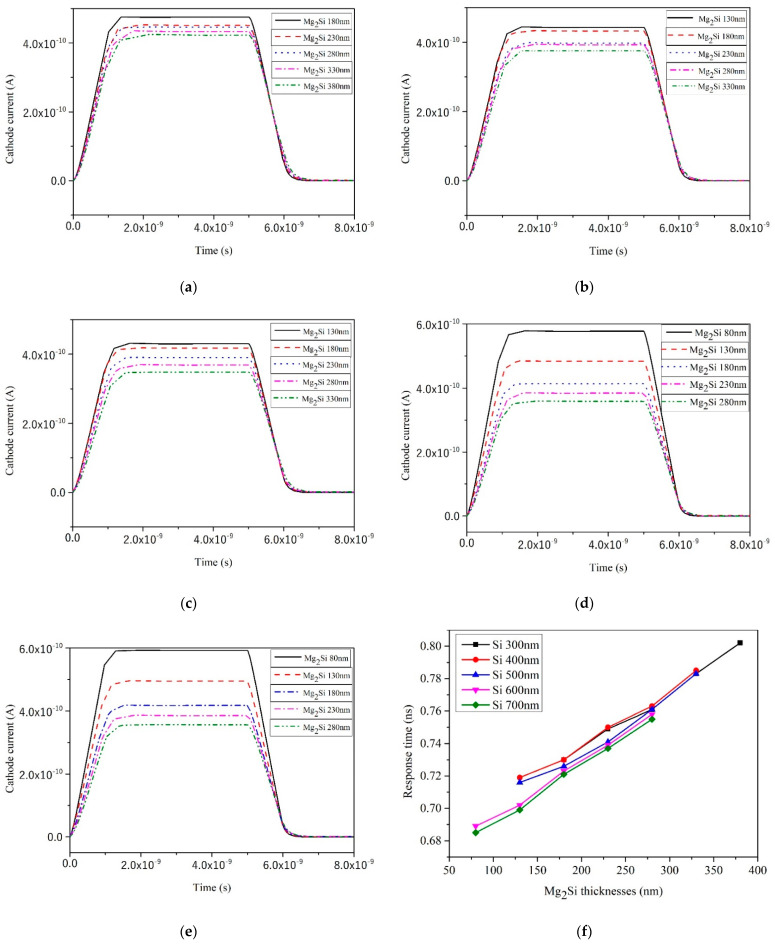

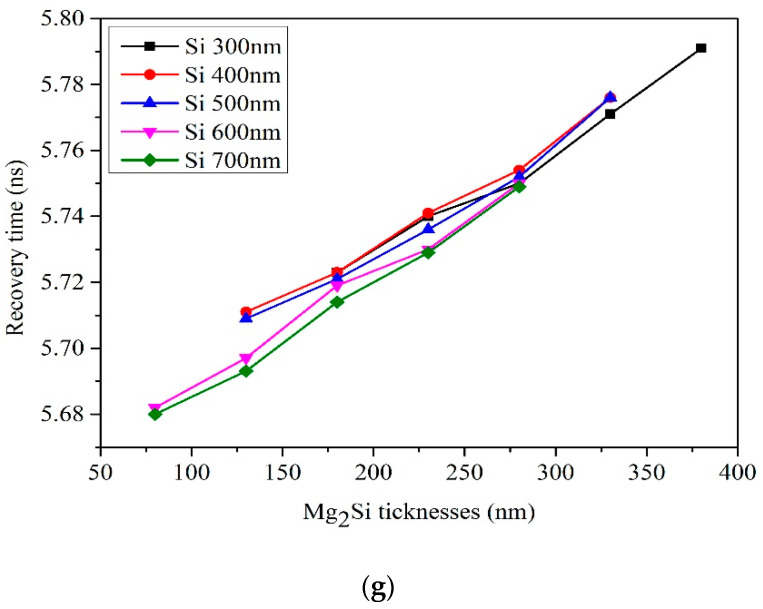
The response time and recovery time curves of heterojunction PD with different Mg_2_Si thicknesses under the condition of 300 nm, 400 nm, 500 nm, 600 nm, and 700 nm thicknesses of Si. (**a**) Si-300 nm; (**b**) Si-400 nm; (**c**) Si-500 nm; (**d**) Si-600 nm; (**e**) Si-700 nm; (**f**) The effect of thicknesses on the response time; (**g**) The effect of thicknesses on the recovery time.

The response time and recovery time curves of heterojunction PD with different thicknesses of Mg_2_Si for Si thicknesses of 300 nm, 400 nm, 500 nm, 600 nm, and 700 nm, respectively, are shown in Figure 12. The response time and recovery time are used to represent the parameters of the transient characteristics of the heterojunction PD. The response time is defined as the time it takes the heterojunction PD to respond from 10% to 90% of the peak value. The time to fall back from 90% of the peak to 10% is the recovery time of the PD [35]. As shown in Figure 12f, the response time values of Mg_2_Si/Si heterojunction PD with different thicknesses are 0.685–0.802 ns. As shown in Figure 12g, the recovery time values of Mg_2_Si/Si heterojunction PD with different thicknesses are 5.68–5.79 ns.

As the thickness of the Mg_2_Si layer increases, both the response time and recovery time of the heterojunction PD increase. This is because the Mg_2_Si layer becomes thicker and longer wavelength light is absorbed, but the long wavelength light needs to penetrate deeper to be fully absorbed [21]. At the same time, under a certain reverse bias voltage and with the increase in Mg_2_Si layer thickness, the resistance of heterojunction PD will decrease, the electric field intensity will increase, the barrier region will expand to a certain extent, and the carrier transit time in the depletion layer will increase [36]. Photogenerated carriers have to experience a long distance from being generated to being swept out by the electric field and the response time will increase. When the light is removed, the photogenerated carriers are completely swept to both ends of the heterojunction by the electric field, which will also take a long time, and the recovery time will increase. With the increase in the thickness of the Si layer, the response time and recovery time show a decreasing trend when the Mg_2_Si layer is thin and an increasing trend when the Mg_2_Si layer is thick. When the thickness of the Mg_2_Si layer is moderate, the change of the response time and recovery time is very small which indicates that the thickness of the Si layer has little influence on the response time and recovery time.

## 4. Conclusions

Mg_2_Si is an environmentally friendly semiconductor material which is non-toxic, harmless, and abundant in Mg and Si. The Mg_2_Si/Si heterojunction PD can detect visible and near-infrared wavelengths and has great potential to be a substitute for other toxic heterojunction PD. In this paper, the model of Mg_2_Si/Si heterojunction photodetectors was built and the performance parameters of Mg_2_Si/Si heterojunction PD with different thicknesses of Mg_2_Si and Si were simulated. The simulation results show that when the thickness of the Si layer is 300 nm and the thickness of the Mg_2_Si layer is 280 nm, the Mg_2_Si/Si heterojunction PD shows optimum performance. For this structure, the following electrical parameters are found: a reverse breakdown voltage of −23.61 V, a forward conduction voltage of 0.51 V, an EQE of 88.98%, a responsivity of 0.437 A/W, an NEP of 6.38 × 10^−12^ WHz^1/2^, a detectivity of 1.567 × 10^11^ Jones, an on/off ratio of 1566, a response time of 0.76 ns, and a recovery time of 5.75 ns. The EQE and responsivity peak wavelength of PD show a redshift as the thickness of Mg_2_Si increases. The Mg_2_Si heterojunction PD shows enormous promise for developing high-performance silicon-compatible PD for efficiently sensing infrared light in the wavelength range of 400 to 1400 nm. Our predictive TCAD simulation results may be utilized as a guideline for the development of future eco-friendly Mg_2_Si/Si heterojunction PD.

## Figures and Tables

**Table 1:** The optical and electrical material parameters for Mg_2_Si and Si.

Parameters and Units	Mg_2_Si [15,16]	Si [16,17,18]
Bandgap (eV)	0.77	1.12
Affinity (eV)	4.37	4.05
Permittivity	20	11.9
Effective conduction band density (cm^−3^)	7.8 × 10^18^	2.8 × 10^19^
Effective valence band density (cm^−3^)	2.06 × 10^19^	1.04 × 10^19^
Electron mobility (cm^2^/V s)	550	1350
Hole mobility (cm^2^/V s)	70	500
Electron auger coefficient (cm^6^/s)	9 × 10^−29^	2.8 × 10^−31^
Hole auger coefficient (cm^6^/s)	9 × 10^−29^	9.9 × 10^−32^
SRH recombination life time, e, h (s)	1 × 10^−6^	1 × 10^−7^

## Data Availability

Data available on request.

## References

[1] Udono H., Yamanaka Y., Uchikoshi M., Isshiki M. (2013). Infrared photoresponse from pn-junction Mg2Si diodes fabricated by thermal diffusion. J. Phys. Chem. Solids.

[2] Zhang Q., Fan J.F., Fan W.H., Zhang H., Chen S.P., Wu Y.C., Tang X.F., Xu B.S. (2020). Energy-Efficient Synthesis and Superior Thermoelectric Performance of Sb-doped Mg_2_Si_0.3_Sn_0.7_ Solid Solutions by Rapid Thermal Explosion. Mater. Res. Bull..

[3] Tamirat A.G., Hou M., Liu Y., Bin D., Sun Y., Fan L., Wang Y., Xia Y. (2018). Highly stable carbon coated Mg2Si intermetallic nanoparticles for lithium-ion battery anode. J. Power Sources.

[4] Pathania A., Madan J., Pandey R., Sharma R. (2020). Effect of structural and temperature variations on perovskite/Mg2Si based monolithic tandem solar cell structure. Appl. Phys. A.

[5] Jin Y.L., Fang H.Z., Wang S., Chen R.R., Su Y.Q., Guo J.J. (2021). Improvement of Microstructure and Mechanical Proper ties of Near-Eutectic Al–Mg_2_Si Alloys by Eu Addition. Adv. Eng. Mater..

[6] Liu J.A., Zhang L.R., Liu S.J., Han Z.W., Dong Z.Q. (2020). Effect of Si content on microstructure and compressive prop erties of open-cell Mg composite foams reinforced by in-situ Mg_2_Si compounds. Mater. Charact..

[7] Shevlyagin A., Chernev I., Galkin N., Gerasimenko A., Gutakovskii A., Hoshida H., Terai Y., Nishikawa N., Ohdaira K. (2020). Probing the Mg_2_Si/Si(111) heterojunction for photovoltaic applications. Sol. Energy.

[8] Masaaki T., Yusuke Y., Masahito U., Haruhiko U. (2013). Spectral characterization of Mg_2_Si pn-junction diode depending on RTA periods. Phys. Status Solidi C.

[9] El-Amir A.A.M., Ohsawa T., Matsushita Y., Wada Y., Shimamura K., Ohashi N. (2018). Preparation and some properties of Mg_2_Si_0.53_Ge_0.47_ single crystal and Mg_2_Si_0.53_Ge_0.47_ pn-junction diode. AIP Adv..

[10] Yu H., Xie Q., Chen Q. (2013). Effects of annealing on the formation of Mg2Si film prepared by resistive thermal evaporation method. J. Mater. Sci. Mater. Electron..

[11] Liao Y.-F., Xie Q., Xiao Q.-Q., Chen Q., Fan M.-H., Xie J., Huang J., Zhang J.-M., Ma R., Wang S.-L. (2017). Photoluminescence of Mg2Si films fabricated by magnetron sputtering. Appl. Surf. Sci..

[12] Yu H., Luo Y., Wang X., He Y., Xu L. (2018). Effects of La doping on Mg2Si semiconductor thin films prepared by thermal evaporation. Mater. Res. Express.

[13] Tan C.L., Jang S.J., Song Y.M., Alameh K., Lee Y.T. (2014). Bimetallic non-alloyed NPs for improving the broadband optical absorption of thin amorphous silicon substrates. Nanoscale Res. Lett..

[14] Karar A., Das N., Tan C.L., Alameh K., Lee Y.T., Karouta F. (2011). High-responsivity plasmonics-based GaAs metal-semiconductor-metal photodetectors. Appl. Phys. Lett..

[15] Kato T., Sago Y., Fujiwara H. (2011). Optoelectronic properties of Mg_2_Si semiconducting layers with high absorption coefficients. J. Appl. Phys..

[16] Deng Q., Wang Z., Wang S., Shao G. (2017). Simulation of planar Si/Mg_2_Si/Si p-i-n heterojunction solar cells for high efficiency. Sol. Energy.

[17] Banhart J., Lay M.D.H., Chang C.S.T., Hill A.J. (2011). Kinetics of natural aging in Al-Mg-Si alloys studied by positron annihilation lifetime spectroscopy. Phys. Rev. B.

[18] Gao Y., Liu H., Lin Y., Shao G. (2011). Computational design of high efficiency FeSi2 thin-film solar cells. Thin Solid Films.

[19] Luo L.B., Wang D., Xie C., Hu J.G., Zhao X.Y., Liang F.X. (2019). PDe_2_ Multilayer on Germanium Nanocones Array with Light Trapping Effect for Sensitive Infrared PD and Image Sensing Application. Adv. Funct. Mater..

[20] Sharma S.N., Sumathi A., Periasamy C. (2017). Photodetection Properties of ZnO/Si Heterojunction Diode: A Simulation Study. IETE Tech. Rev..

[21] Yang C., Liang H., Zhang Z., Xia X., Tao P., Chen Y., Zhang H., Shen R., Luo Y., Du G. (2018). Self-powered SBD solar-blind photodetector fabricated on the single crystal of β-Ga_2_O_3_. RSC Adv..

[22] Xie C., Zeng L.H., Zhang Z.X., Tsang Y.H., Luo L.B., Lee J.H. (2018). High-performance broadband heterojunction PD based on multilayered PtSe_2_ directly grown on Si substrate. Nanoscale.

[23] Liu K.W., Makoto S., Masakazu A. (2010). ZnO-Based Ultraviolet PD. Sensors.

[24] Zhu M., Zhang L., Li X., He Y., Li X., Guo F., Zang X., Wang K., Xie D., Li X. (2015). TiO_2_ enhanced ultraviolet detection based on a graphene/Si Schottky diode. J. Mater. Chem. A.

[25] Zhu M., Li X., Chung S., Zhao L., Li X., Zang X., Wang K., Wei J., Zhong M., Zhou K. (2015). Photo-induced selective gas detection based on reduced graphene oxide/Si Schottky diode. Carbon.

[26] An X., Liu F., Jung Y.J., Kar S. (2013). Tunable Graphene–Silicon Heterojunctions for Ultrasensitive Photodetection. Nano Lett..

[27] Es-Souni M., Zhang N., Iakovlev S., Solterbeck C.-H., Piorra A. (2003). Thickness and erbium doping effects on the electrical properties of lead zirconate titanate thin films. Thin Solid Films.

[28] Li G., Li Z.Q., Chen J.W., Chen X., Qiao S., Wang S.F., Xu Y., Mai Y.H. (2017). Self-powered, high-speed Sb2Se3/Si het erojunction PD with close spaced sublimation processed Sb_2_Se_3_ layer. J. Alloy. Compd..

[29] Xu J., Zhang H., Song Z., Xu Y., Peng Q., Xiu X., Li Z., Li C., Liu M., Man B. (2020). SnS_2_/Si vertical heterostructure for high-performance photodetection with large photocurrent and fast speed. Appl. Surf. Sci..

[30] Hun C.M., Tien C.H., Lee K.L., Lai H.Y., Chen L.C. (2021). The Effects of Temperature on the Growth of a Lead-Free Perovskite-Like (CH_3_NH_3_)_3_Sb_2_Br_9_ Single Crystal for an MSM PD Application. Sensors.

[31] Liang F.X., Zhao X.Y., Jiang J.J., Hu J.G., Xie W.Q., Lv J., Zhang Z.X., Wu D., Luo L.B. (2019). Light Confinement Effect Induced Highly Sensitive, Self-Driven Near-Infrared PD and Image Sensor Based on Multilayer PDe_2_/Pyramid Si Heterojunction. Small.

[32] Luo L.B., Zhang X.X., Li C., Li J.X., Zhao X.Y., Zhang Z.X., Chen H.Y., Wu D., Liang F.X. (2020). Fabrication of PDe_2_/GaAs heterojunction for sensitive near-infrared photovoltaic detector and image sensor application. Chin. J. Chem. Phys..

[33] Hong Q.S., Cao Y., Xu J., Lu H.M., He J.H., Sun J.L. (2014). Self-Powered Ultrafast Broadband PD Based on p−n Heterojunctions of CuO/Si Nanowire Array. ACS Appl. Mater. Interfaces.

[34] Li X.X., Sun T., Zhou K., Hong X., Tang X.Y., Wei D.C., Feng W.L., Shen J., Wei D.P. (2020). Broadband InSb/Si het erojunction PD with graphene transparent electrode. Nanotechnology.

[35] Das B., Das N.S., Sarkar S., Chatterjee B.K., Chattopadhyay K.K. (2017). Topological insulator Bi_2_Se_3_/Si nanowires based pn junction diode for high performance near infrared PD. ACS Appl. Mater. Interfaces.

[36] Lu Z., Chen Z.M., Pu H.B. (2005). SiCGe/SiC Heterojunction and Its MEDICI Simulation of Optoelectronic Characteristics. Chin. Phys. B.

